# Does Malleolus non-Lifting Tympanoplasty have any Advantage Over Malleus Lifting Techniques?

**Published:** 2016-01

**Authors:** Mohammad Reza Vahidi, Abolfazl Mollasadeghi, Honeyeh Shahbazian, Nasim Behniafard, Mohammad Hossein Dadgarnia

**Affiliations:** 1 *Otorhinolaryngology Research Center, Shahid Sadoughi University of Medical Sciences, Yazd, Iran.*; 2 *Department of Occupational Medicine, Shahid Sadoughi University of Medical Sciences, Yazd, Iran.*

**Keywords:** Auditory Threshold, Hearing loss, Lifting, Otitis media, Tympanoplasty

## Abstract

**Introduction:**

In order to achieve a higher success rate for tympanoplasty, different techniques have been developed, and a wide variety of grafting materials have been developed. One of the techniques currently receiving considerable attention involves not lifting the remaining of eardrum from the malleus and embedding the graft underneath in order to repair the eardrum correctly in its original position, as well as minimizing graft lateralization leading to progression of hearing rehabilitation. We compared the effects of tympanoplasty with and without malleus lifting on hearing loss in patients with chronic otitis media.

**Materials and Methods:**

In this study, 30 consecutive patients diagnosed as having chronic otitis media without cholesteatoma were randomly assigned to two tympanoplasty groups; with or without malleus lifting. Air and bone conduction thresholds were recorded before and 45 days after the intervention.

**Results:**

In groups, except for 8000 Hz, the air conduction was significantly improved following surgery. According to air conduction there was no difference between the groups before surgery at different frequencies, although it was improved to a greater degree in the group without lifting at 250 Hz postoperatively. The average post-operative air-bone gap (ABG) gain was significantly higher in all study frequencies in the target group. One of the effects of this technique is inner-ear protection from physical trauma to the ossicular chain, and prevention of damage to bone conduction.

**Conclusion:**

A higher hearing threshold and also higher ABG gain can be achieved by not lifting the remaining eardrum from the malleus and embedding the graft undereath it, especially at lower frequencies.

## Introduction

Otitis media (OM) is an important health problem worldwide, leading to both structural and psychological sequelae (1). OM is an inflammatory-based disorder with or without an infectious cause that can appear as acute or chronic, suppurative or non-supportive, and serous or secretory (2). Progressing OM, especially in chronic conditions, may lead to tympanic membrane perforation and hearing loss (3). The severity of this complication is dependent on a variety of disease-related factors, including the size and position of the tympanic perforation, the degree of membrane and ossicles fixation, ossicles erosion, ossicular chain disruption and the degree of repercussion in the inner ear (4,5). 

Although OM can be successfully treated in the early stages via non-invasive medical methods, in more deteriorating stages, OM complications, especially hearing loss, should be managed through surgical techniques such as tympanoplasty with middle ear and ossicular chain exploration, and tympanic membrane reconstruction (6-8). The main goal of tympanoplasty is to re-establish sound protection which results in improving sound transmission and thus protecting against hearing process. In tympanoplasty, defects in the tympanic membrane can be repaired by replacing a graft, either medial or lateral to the tympanic membrane annulus (9). This technique can be successful following eradication of inflamed granulation tissue and cholesteatoma from the middle ear (10). To achieve higher tympanoplasty effectiveness, different techniques have evolved and have been refined, and a number of grafting materials are available. Today, one of the techniques receiving considerable attention involves not lifting the remaining eardrum from the malleus and embedding the graft underneath it, resulting in repairing the eardrum correctly in its original position as well as minimizing graft lateralization, leading to progression of hearing rehabilitation (11,12). However, the success rate of this new technique remains controversial. The present study aimed to assess and compare the effects of tympanoplasty with and without malleus lifting on hearing loss in patients suffering from chronic OM. 

## Materials and Methods

*Study Population:*

Thirty consecutive patients diagnosed as having chronic OM without cholesteatoma were included in this randomized controlled trial. These patients were selected consecutively in two hospital clinics of Shahid Sadoughi University of Medical Sciences (Shahid Sadoughi and Shahid Rahnemun hospital clinics) from June 2012 to June 2014. The main exclusion criteria included the presence of cholesteatoma or other OM-related complications affecting hearing, a history of recent usage of ototoxic medications or any types of previous otologic surgeries in each side, a history of Menier's disease or any kind of systemic disease including psychological disorders, and also being exposed to intense noise on the day of admission and evaluation. 

This study was approved by the Ethics Committee of Research Vice-Chancellor of the Shahid Sadoughi University of Medical Sciences, Yazd, Iran. Written consent forms were signed by patients giving their informed participation in the present study (in Persian). 

*Intervention Protocol:*

Baseline characteristics, including demographic information, medical history, medication, and occupational state were collected on admission via an interview. At initial assessment, all subjects underwent a physical examination including assessment of pure tone audiometry (PTA) performed using air conduction and bone conduction modes (device: AC40, Interacoustic, Denmark, headphone: TD39). Then, patients were randomly allocated into one of two groups **using** a computerized random-number generator immediately prior to the procedure (tympanoplasty with and without malleus lifting). All procedures were conducted by a single surgeon. After 45 days following the procedure, the level of hearing and the patients’ satisfaction with the results of surgery were recorded in both groups, and all patients were examined with regard to the quality of the surgical repair of the perforation of the tympanic membrane by audiometry. The air and bone conduction thresholds were recorded before and 45 days after the intervention. The average of air conduction and bone conduction thresholds were calculated by taking the averages of 250, 500, 1000, 2000, 4000, and 8000 Hz frequencies. Air-bone gap (ABG) was calculated in accordance with the American Academy of Otolaryngology-Head and Neck Surgery (AAO-HNS)1995guidelines (13).

The surgical outcome was compared in each group before and after the surgery, and also between the study groups. For statistical analysis, results are presented as mean ± standard deviation (SD) for quantitative variables and are summarized by frequency (percentage) for categorical variables. Continuous variables were compared using a t-test and Mann-Whitney U test whenever the data did not appear to have normal distribution or when the assumption of equal variances was violated across the study groups. Categorical variables were compared using the chi-square test. The changes in study parameters before and after surgical repair were compared using a paired t-test or **Wilcoxon** signed-rank **test.** For statistical analysis, SPSS version 16.0 for windows (SPSS Inc., Chicago, IL) was used. P-values of 0.05 or less were considered statistically significant.

## Results

The average air conduction thresholds before and after intervention in both groups are presented in (Table. 1). In both groups, except for 8000 Hz, the air conduction was significantly improved following surgery. Comparing air conduction between the groups with and without malleus lifting, no difference was found between the groups before surgery at the different frequencies. 

**Table 1:** Comparing air conduction thresholds between the groups with and without malleus lifting

	Before			After		
	With lifting	Without lifting	P-value	With lifting	Without lifting	P-value
**AC250**	56.28±13.94	52.86±15.46	0.386	24.49±11.74	33.33±16.53	0.019
**AC500**	48.72±15.25	47.86±18.00	0.846	24.87±11.38	31.19±15.88	0.080
**AC1000**	49.62±16.36	47.38±17.72	0.626	25.64±13.82	31.19±16.65	0.173
**AC2000**	54.10±18.95	49.52±19.68	0.382	32.05±20.16	35.24±20.03	0.561
**AC4000**	62.69±22.85	56.90±21.59	0.344	45.38±21.62	47.14±22.06	0.767
**AC8000**	58.97±24.66	54.05±26.91	0.477	63.97±30.31	70.24±25.27	0.216

**Fig 1:**
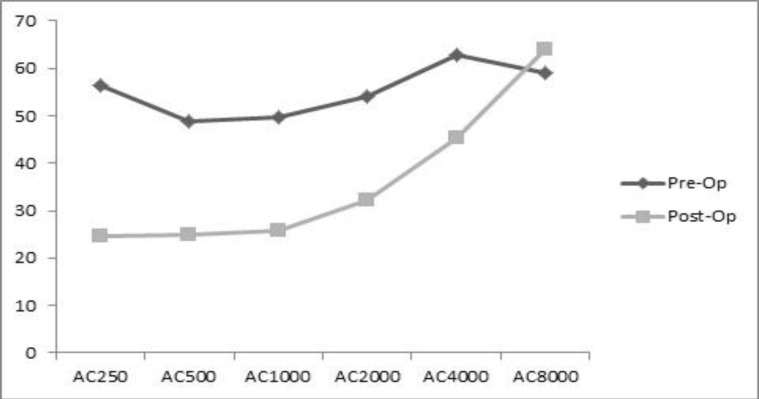
Air conduction before and after surgery in group without malleus lifting

**Fig 2:**
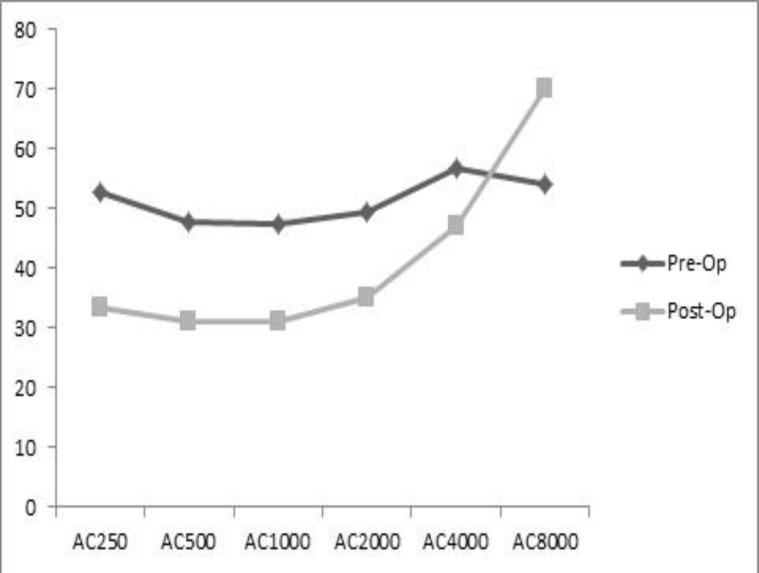
Air conduction before and after surgery in group with malleus lifting

Figures 1 and 2 compare the air conduction thresholds in each group before and after intervention (Figs 1,2).

The average post-operative PTA-ABG gain was significantly higher in all study frequencies in the group without malleus lifting compared with the control group. In this regard, the average ABG gain was 31.79 dB and 19.53 dB at 250 Hz (P<0.001), 23.85 dB and 16.67 dB at 500 Hz (P=0.012), 23.98 dB and 16.19 dB at 1,000 Hz (P=0.004), and 22.05 dB and 14.28 dB at 2000 Hz (P=0.039), but not at frequency of 4,000 Hz (17.31 dB and 9.76 dB, respectively, P= 0.083). The results of this study showed that in the lifting group after surgery, the bone conduction significantly decreased only at 4,000 Hz (P<0001), while in the non-lifting group, after surgery the bone conduction in all of the frequencies showed no change (P=0.1). One of the effects of this technique is inner-ear protection from physical trauma to the ossicular chain, and prevention of damage to bone conduction.

## Discussion

The current study was performed to compare pre- and post-operative hearing results in terms of hearing threshold and PTA-ABG. We describe our experience on a new surgical alternative in which the remainder of the eardrum is not lifted from malleus and the graft is embedded under it. We hypothesized that the procedure employed may lead to successful repair of the eardrum, in its original place, and also allow minimization of graft lateralization leading to greater improvement of hearing threshold. 

We have shown that, although both techniques (with and without malleus lifting) improved ABG, this improvement was more pronounced in the latter group. On the other hand, not only can the latter technique minimize graft lateralization, it can also improve the hearing threshold. However, it should be noted that the marked improvement in hearing threshold by the new technique is more pronounced at lower frequencies. In this regard, the average ABG gain was achieved at frequencies less than 4000 Hz. Therefore, along with the technical success of improvement of hearing threshold, it seems that the technique used can prevent worsening of bone conduction and cartilage extrusion. 

Some previous studies support our findings. In a study by Stage et al. (14) on surgical outcomes after underlay tympanoplasty, it was shown that the hearing threshold in grafts lateral to the malleus was comparable when the grafts were medial to the malleus. Kutluhan et al. (15) also assessed the effects of resection of the tip of the manubrium mallei on the success rate of tympanoplasty and indicated that the pointed technique could lower surgical complications. To the best of our knowledge, there are no other studies inthis area. 

In this study, we assessed the short-term surgical results, because the long-term consequences of any ossicular repairing are largely dependent on several factors beyond the surgeons’ control, such as follow-up rate of patients, probable exposure to intense noise, middle ear stability, and the condition of the mucosa.

 Also, one of the effects of this technique is inner-ear protection from physical trauma to the ossicular chain, and prevention of damage to bone conduction. These short-term results may accurately reflect the actual reconstructive procedures. However, the patients’ satisfaction should be assessed over longer follow-up times and also when applying larger sample sizes.

## Conclusion

In conclusion, our study showed that not lifting the remaining of eardrum from the malleus and embedding the graft under it leads to a higher hearing threshold and also higher ABG gain, especially in the lower frequencies. 
